# Seasonal patterns of tuberculosis case notification in the tropics of Africa: A six-year trend analysis in Ethiopia

**DOI:** 10.1371/journal.pone.0207552

**Published:** 2018-11-26

**Authors:** Z. Gashu, D. Jerene, D. G. Datiko, N. Hiruy, S. Negash, K. Melkieneh, D. Bekele, G. Nigussie, P. G. Suarez, A. Hadgu

**Affiliations:** 1 Management Sciences for Health, USAID Challenge TB Project, Addis Ababa, Ethiopia; 2 Oromia Regional Health Bureau, Addis Ababa, Ethiopia; 3 Amhara Regional Health Bureau, Addis Ababa, Ethiopia; 4 Management Sciences for Health, Arlington, Virginia, United States of America; University of Liverpool, UNITED KINGDOM

## Abstract

**Objective:**

Seasonal variations affect the health system’s functioning, including tuberculosis (TB) services, but there is little evidence about seasonal variations in TB case notification in tropical countries, including Ethiopia. This study sought to fill this gap in knowledge using TB data reported from 10 zones, 5 each from Amhara and Oromia regions.

**Methods:**

Notified TB cases for 2010–2016 were analyzed using SPSS version 20. We calculated the quarterly and annual average TB case notification rates and the proportion of seasonal amplitudes. We applied Winters’ multiplicative method of exponential smoothing to break down the original time series into seasonal, trend, and irregular components and to build a suitable model for forecasting.

**Results:**

A total of 205,575 TB cases were identified (47.8% from Amhara, 52.2% from Oromia), with a male-to-female ratio of 1.2:1. The means of 8,200 (24%), 7,992 (23%), 8,849 (26%), and 9,222 (27%) TB cases were reported during July-September, October-December, January-March, and April-June, respectively. The seasonal component of our model indicated a peak in April-June and a trough in October-December. The seasonal amplitude in Amhara region is 10% greater than that of Oromia (p < 0.05).

**Conclusions:**

TB is shown to be a seasonal disease in Ethiopia, with a peak in quarter four and a low in quarter two of the fiscal year. The peak TB case notification rate corresponds with the end of the dry season in the two agrarian regions of Ethiopia. TB prevention and control interventions, such as efforts to increase community TB awareness about TB transmission and contact tracing, should consider seasonal variation. Regional variations in TB seasonality may require consideration of geographic-specific TB case-finding strategies. The mechanisms underlying the seasonal variation of TB are complex, and further study is needed.

## Introduction

Evidence of seasonal variation in TB caseloads from countries in the northern temperate zone exists [1–14]. These studies have indicated that TB case notifications reach a peak in late spring or summer and fall to their lowest point in winter. Plausible explanations include the relatively short, dark days and hence low vitamin D in the blood during the winter that leads to active TB disease during the summer [4,12–16]; overcrowding and indoor activities during winter, enhancing indoor transmission of TB that becomes symptomatic in summer [12,13,15]; and lower cell-mediated immunity during the winter, leading to TB reactivation during summer [1]. Seasonal variation in food availability and food intake may also play a role in the variability of TB notification [16].

In the tropics, unlike the temperate zone, day and night are of relatively equivalent length. Studies from semi-tropical countries such as India [17–18], Pakistan [19], and Cameroon [20] have shown that TB notification is high during summer and low during winter.

Ethiopia is among the 30 high TB, TB/HIV and multidrug-resistant-TB (MDR-TB) burden countries [21]. The TB case notification rate in Ethiopia has declined by 8% per year from a peak in 2011 [22]. The respective annual estimated TB incidence and death rates per 100,000 population were 177 and 25 for 2016 [22]. In 2016, Ethiopia’s case notification for new and relapse TB cases was 125,836 [22]. Thus, the country has not notified the number of cases estimated by the World Health Organization [22] and has continued to miss about a third of the annual number of TB cases [21–22]. To narrow the gap between the expected and actual reported TB cases, the National TB Program has been implementing a comprehensive TB case-finding strategy, including community TB care, targeted interventions among key population groups, routine TB screening in health facilities, and improved diagnostic capacity.

Understanding the epidemiology of TB in the country in terms of seasonal variation would make significant contributions to designing high-yield case-finding strategies. A few studies in Ethiopia have tried to indicate the seasonal variation in TB, but their findings are inconsistent and limited in the scope. A study done at the district level in northern Ethiopia showed that about 60% of the smear-positive TB cases were detected in the dry season of the year (defined as December-February) [23]. A community-based study in the southern part of the country indicated that the case detection rate of TB was low in autumn (March-May) and high in early summer (June-August) [24]. However, about 90% of the geographic locations in Ethiopia experience a heavy rainy season from June to September and a dry season from October to April [25], so a more precise and broader study is warranted to assess the seasonal patterns of TB. Hence, this study aimed to determine seasonal variations in TB caseload and provide evidence for national policymakers and decision-makers.

## Methods

### Study design and setting

Ethiopia is the second most populous nation in Africa, with a population of about 104 million living in nine administrative regions and two federal cities [25]. Oromia and Amhara are the two agrarian regions and the largest regions in the country with a total population of 62.4 million, about 60% of the national estimate.

Amara is located in the north western and north central part of Ethiopia, where about 81.5% of the residents are Orthodox Christians and about 85% engage in agriculture. The region has both the highlands and lowlands. The highlands are above 1500 meters above sea level and comprise the largest part of the northern and eastern parts of the region. The lowland part covers mainly the western and eastern parts with an altitude between 500–1500 meters above sea level. The annual mean temperature in most parts of the Amhara region lies between 15°C-21°C and receives 80% of the total rainfall in the country during the summer season, mid-June to September [26].

The Oromia region sprawls over the 32% of the surface area of the country, where about 44.3% of the dwellers are Muslims and over 90% of them live in the rural area living on agriculture. There are high and rugged mountain ranges rising from less than 500 meters above sea level to high ranges that culminate up to 4607 meters in the region [27].

The case notification rates per 100,000 population in Oromia and Amhara were 119 and 106, respectively, in 2016 [28]. More than half of all forms of TB cases reported in the country came from these two regions [22, 28]. Administratively, a region in Ethiopia is divided into zones, and a zone is subdivided into districts or *woredas*. The smallest administrative unit is called a *kebele*. The Ethiopian fiscal year is divided into four quarters; quarter one (July-September), quarter two (October-December), quarter three (January-March) and quarter four (April-June).

The study was based on TB reports from health facilities in 10 zones, 5 zones each from the Amhara and Oromia regions through projects funded by the US Agency for International Development (map S1 Fig). For this study, data from 24 quarters for all forms of TB was collected from July 2010 to June 2016. Ethiopia uses a quarterly reporting mechanism that starts in July in a fiscal year.

During the study period, TB was diagnosed by Ziehl-Neelsen microscopy using spot and early morning sputum samples. The presence of *M*. *tuberculosis* in two sputum samples in HIV-negative individuals and one in HIV-positive individuals was categorized as smear-positive pulmonary TB (SS+); whereas diagnosis by chest X-ray or decision by a clinician when all three sputum samples were negative was classified as smear-negative pulmonary TB (SS−). Extrapulmonary TB (EPTB) was TB outside the lungs, diagnosed based on clinical manifestation, clinical decision, radiography, or histopathology [29]. The reports used in this study contained TB cases diagnosed before the introduction of GeneXpert.

### Data quality assurance and analysis

Data quality assurance (DQA) and supportive supervision on TB diagnosis were carried out quarterly. A quarterly cycle was assumed to establish a seasonal pattern. Excel data was exported to SPSS version 20 and analyzed. We computed average TB case notification rates with a 95% confidence interval (CI). We also plotted a line graph using the unadjusted number of quarterly TB cases detected and the quarterly differences. The original time series of total TB cases was broken down into seasonal, trend, and irregular components using a multiplicative seasonal decomposition method. The isolated trend cycle curve was smoothed after excluding the seasonal and irregular variations. The trend components reflect the long-term progression of the series, the seasonal components describe seasonal variation, and the irregular components reflect random and irregular influences.

The Winters multiplicative method of exponential smoothing was used to forecast time series. According to the multiplicative exponential smoothing, the Ljung-Box test was correctly specified and there was no outlier in the data (S1 Table). The stationary squared value was used to estimate the proportion of the total variation in trend and seasonal pattern. The stationary value explained about 46.1% of the observed variation in the overall series, 66.5% in Oromia and 42.1% in Amara (S1 Table). The stationary squared value of the data was checked by the autocorrelation function and partial autocorrelation function.

The proportion of seasonal amplitude for TB disease, the mean peak quarter, mean trough quarter, and annual seasonal amplitude were calculated with 95% CI. Seasonal amplitude is a proportion of the highest-to-the lowest difference of average TB cases between the quarter with the peak and the quarter with the trough, and the denominator is the average TB cases for the year. We did a t-test for categorical variables. A p value < 0.05 was considered significant.

### Ethical considerations

The Ethics Review Committees of Oromia and Amhara Regional Health Bureaus reviewed and approved the study protocol. The letter of permission was written to the 10 zones to access to their quarterly reports. The data from the reports did not contain personal identifying information.

## Results

Over the six years, 205,575 TB cases were notified, 47.8% from Amhara and 52.2% from Oromia. Females constituted 45%. The proportions of SS+, SS−, and EPTB cases were 20.2%, 31.9%, and 38.9%, respectively. About 12% were children below 15 years of age (data in S2 Table). The mean of 8,566 TB cases were reported every quarter, with the highest number of cases in the fourth quarter and the lowest in the second quarter (Table 1).

**Table 1:** Quarterly summary of total TB cases from July 2010 through June 2016.

Quarters	Total TB Cases	Quarterly Proportion (%)	Mean	Mean Standard Error	95% CI of the Means
Quarter 1 (Q1)	49197	23.9	8199.5	269.9	7505.7–8893.3
Quarter 2 (Q2)	47951	23.3	7991.8	90.7	7758.6–8225.1
Quarter 3 (Q3)	53094	25.8	8849	175.6	8397.6–9300.4
Quarter 4 (Q4)	55333	26.9	9222.2	295.9	8461.5–9982.9
**Total**	**205575**	**100**	**8565.6**	**146.4**	**8262.8–8868.5**

**Fig 1:**
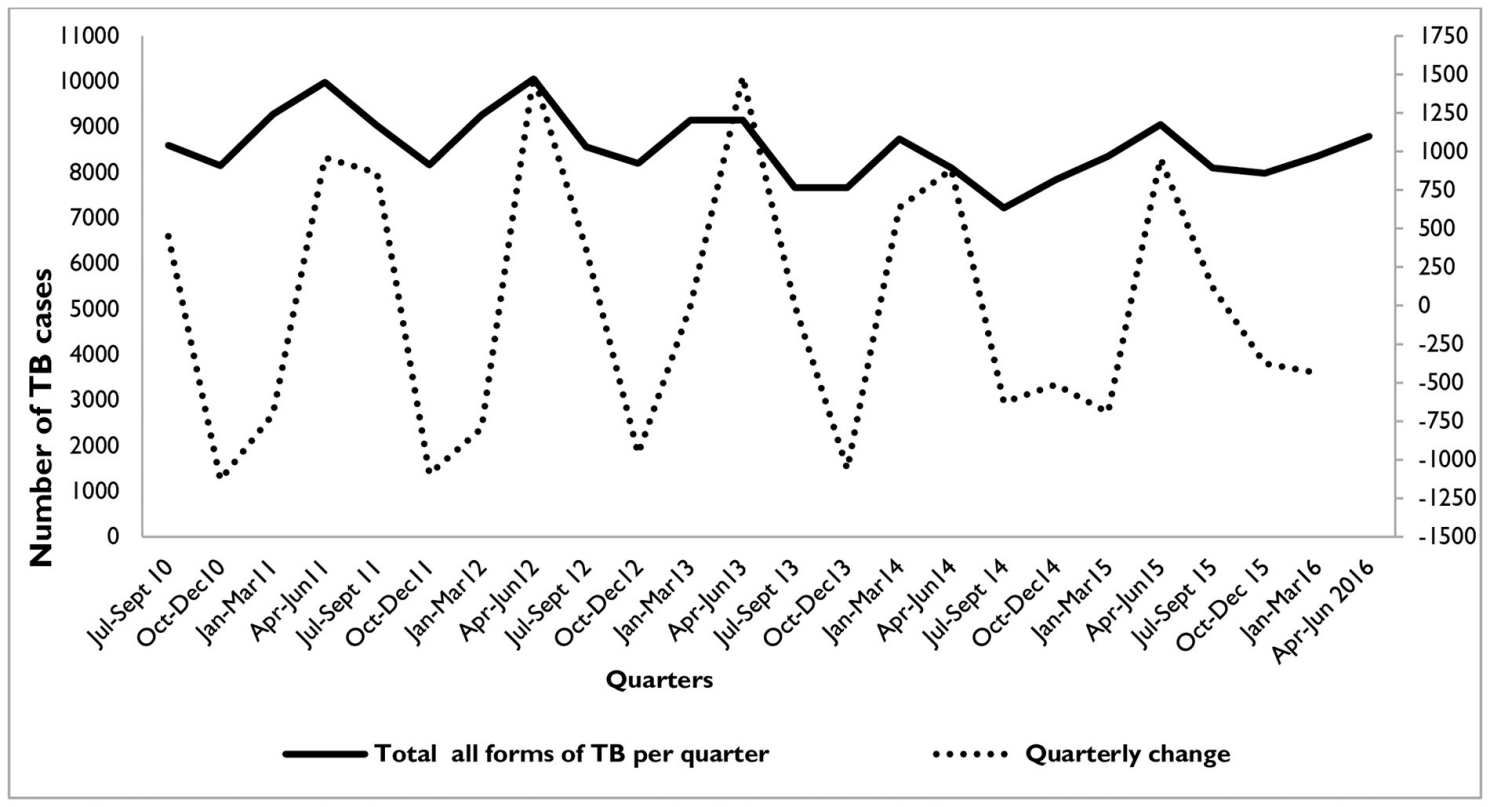
Seasonal trends of TB case notified from agrarian regions of Ethiopia, July 2010-June 2016. **Months**: Jul = July; Sep = September; Oct = October; Jan = January; Mar = March; Apr = April; Jun = June.

Fig 1 shows the quarterly pattern and the difference from the observed data where the number of notified TB cases started to increase during January to March, reaching a peak between April and June. The caseload started to decrease during the quarter from July to September. The lowest number of TB cases was reported during the period October to December. This cyclical pattern was observed throughout the six years.

**Fig 2:**
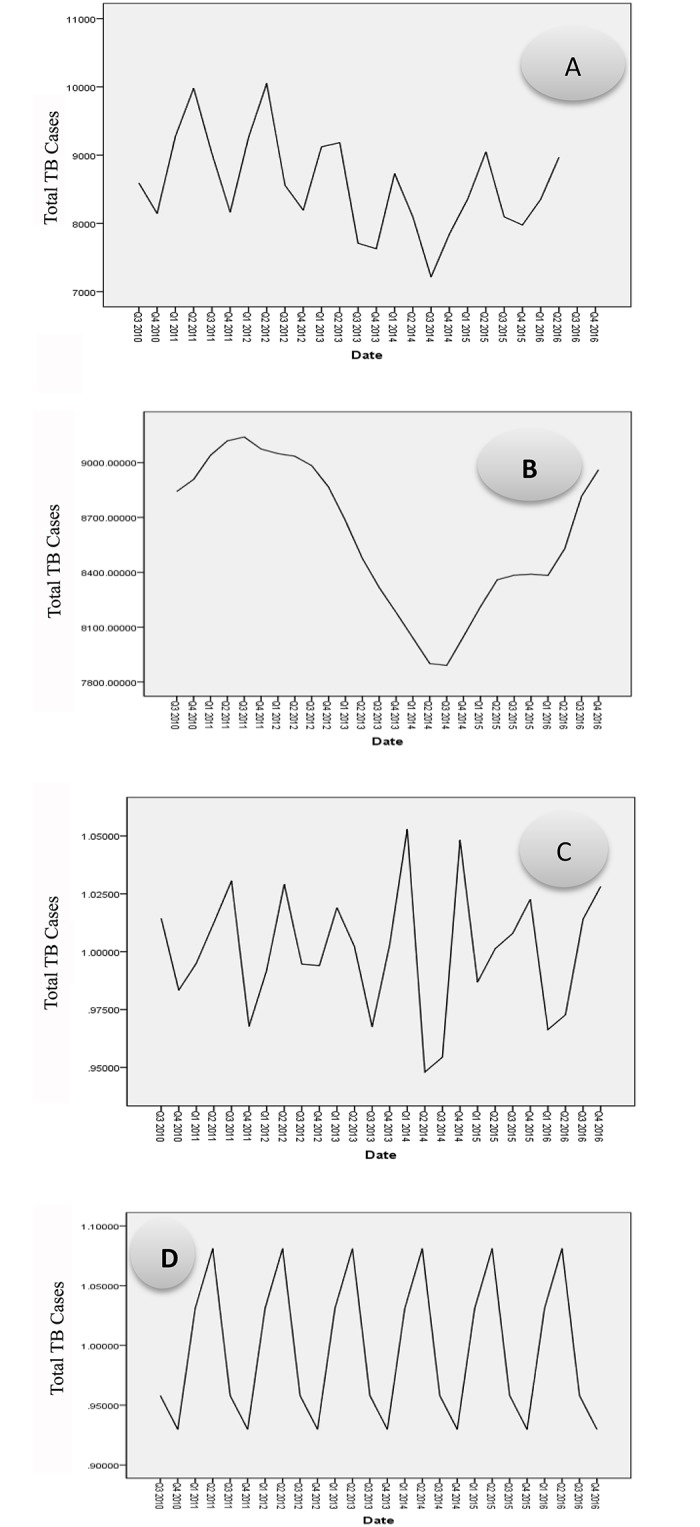
A sequence chart for original series and trend, seasonal, and irregular components,2010–2016. **Quarters**: Q1 is the first quarter (July-September); Q2 is the second quarter (October-December); Q3 is the third quarter (January-March) and Q4 is the fourth quarter (April-June) **A**. the original quarterly time series; **B**. is the decomposed time series into the trend cycle; **C**. is a seasonal trend; and **D**. is an irregular components.

A regular annual cyclical and seasonal fluctuation of TB cases is demonstrated by the original quarterly time series (Fig 2A). This series was decomposed into the trend cycle (Fig 2B), seasonal (Fig 2C), and irregular components (Fig 2D). Until quarter three of 2014, the isolated trend was declining, followed by an increasing trend until 2016. The seasonality components showed a peak in April to June and the lowest record in October to December.

**Fig 3:**
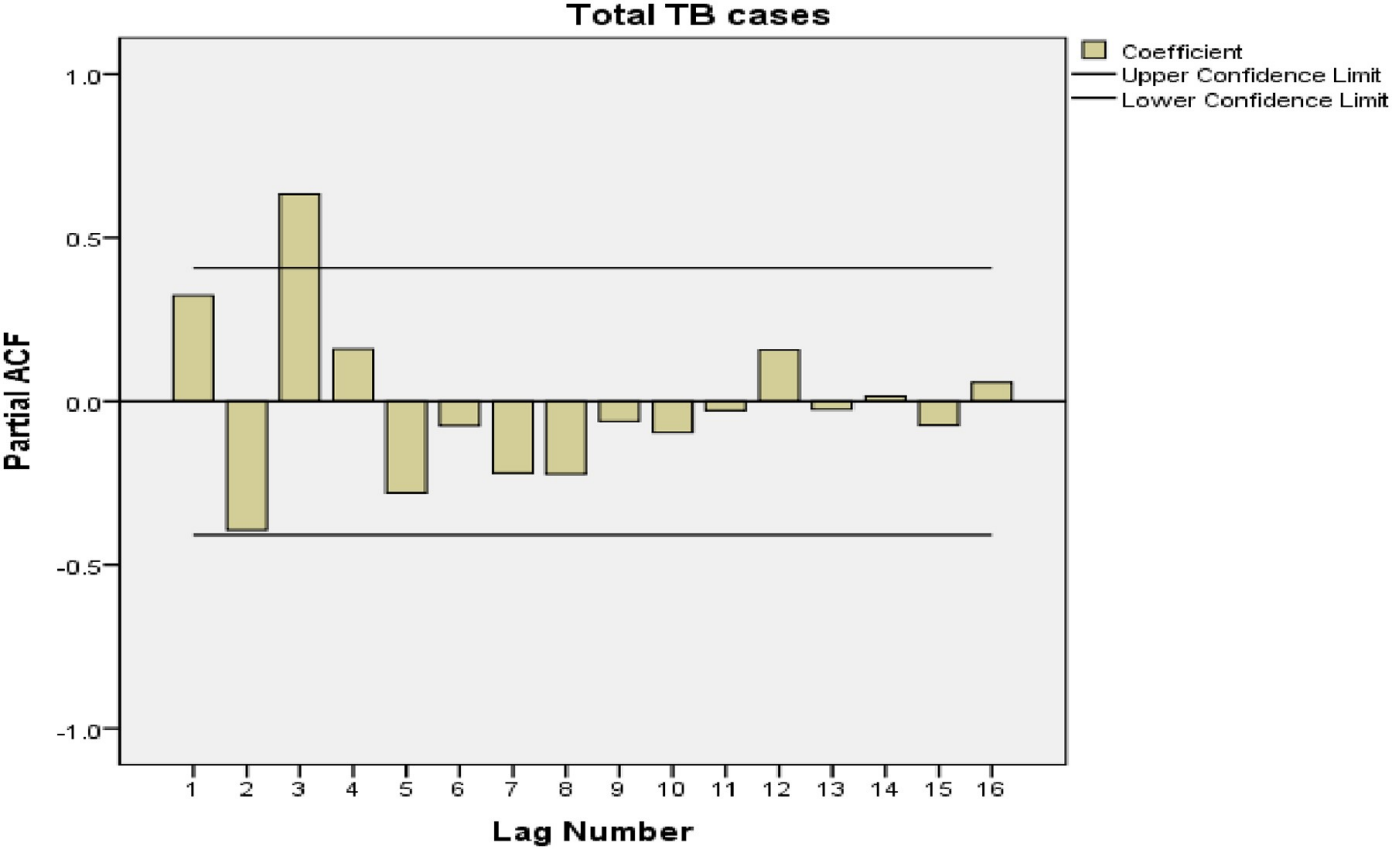
PAF of the quarterly notified TB cases from 2010–2016 with a peak at a lag of 3. ACF: autocorrelation function; PAF: Partial autocorrelation function.

A significant peak was observed at a lag of 4 (autocorrelation factor = 0.613; Ljung-Box statistic [p = 0.001]). This indicates the presence of seasonality in this data. The partial autocorrelation factor showed a significant peak at a lag of 3 as well. This confirms the presence of the seasonal component (Fig 3).

The yearly seasonal amplitude for total TB cases was 16.8% (11.5–22.02%). This reveals an annual mean of 16.8% more cases of TB diagnosed in the peak quarter (quarter four) as compared to the lowest quarter (quarter two). The greatest (23.1%) and the lowest (13.3%) seasonal amplitudes were found in Amhara and Oromia regions, respectively. Seasonal amplitude in Amhara Region is greater by 10% as compared to seasonal amplitude in Oromia Region (p < 0.05) (Table 2).

**Table 2:** Comparison of seasonal amplitude of TB cases among different groups.

Variables	Mean Difference (Peak-Trough)	Mean Seasonal Amplitude (%)	SE of Seasonal Amplitude	95% CI	Mean Comparisons	T-value	P-value
Total TB cases	1440.8	16.8	2	11.5–22.0			
Total SS+	383.7	15.3	2.3	9.3–21	Total SS+ & total EPTB	−2.1	0.9
Total EPTB	591	17.6	2.1	12.3–22.9			
Total SS−	461.5	16.9	2	11.8–22.1			
Male	787.7	16.7	2.1	11.3–22.0	Male & female	1	0.2
Female	653.2	16.9	2	11.7–22.1			
Oromia	587.5	13.3	2.9	5.9–20.7	Oromia & Amhara	2.2	0.001
Amhara	951.3	23.1	3.4	14.5–31.8			
>15 years	1270.3	16.9	2.1	11.6–22.1	>15 years & <15 years	0.2	0.4
<15 years	170.5	16.3	2.1	10.8–21.7			
<5 years	30.7	15	2.6	8.3–21.6	>15 years & <5 years	0.6	0.3

**Fig 4:**
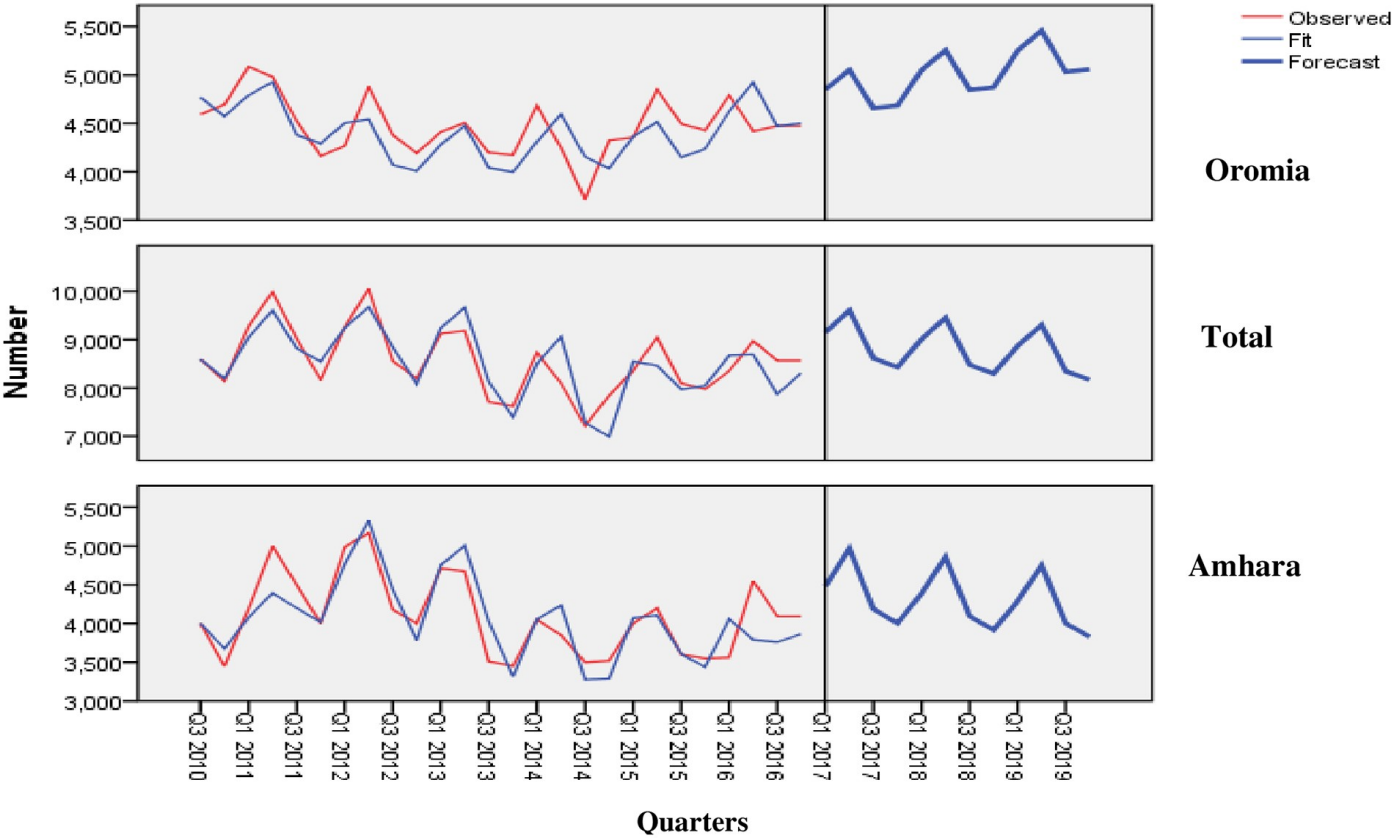
A seasonal time series TB case notification and forecast of Amhara, Oromia and the total.

To forecast the TB case notification for the future, the exponential smoothing model with Winters’ multiplicative method was utilized from quarter three of 2016 to quarter three of 2019. The Ljung-Box goodness-of-fit test revealed that there was no significant (p > 0.1) departure of the observed frequencies from the fitted seasonal pattern (S1 Table). It showed the seasonally cyclical pattern of TB notification in both regions. There is also a declining trend in Amhara, an increasing trend in Oromia, and a relatively stable pattern in the overall number of TB cases notified (Fig 4).

## Discussion

We observed seasonal variation of TB in Ethiopia, with the highest case notification rate seen in April-June (the end of the dry season) and the lowest notification rate during October-December (the beginning of the dry season). Amhara has a higher seasonal amplitude than Oromia. We also observed an increasing overall case notification trend in Oromia and a decreasing trend in Amhara. The findings suggest the need to explore season-specific approaches for case finding in Ethiopia.

The seasonal variations in the numbers of TB cases notified in this study correspond to the results of other studies [4–6,8, 10]. The most plausible explanations for temperate zones were greater risk of transmission of *M*. *tuberculosis* during the winter months due to indoor activities, seasonal change in immune function, delays in the diagnosis and treatment of TB, periodic variation in food availability and food intake, age, and sex [1].

Seasonal rainfall and humidity and workload have been observed to influence the time of disease peak [20,30]. Likewise, in our study, the wet or rainy season is likely to affect attendance at diagnostic or outpatient services in health facilities [30]. Rural agrarian populations of Ethiopia carry out their major farming activities from June to September, with the harvest season taking place in October to March. In addition, the rural population has low income during the farming and harvesting periods. Therefore, visits to health facilities for chronic illnesses like TB may be fewer. People might display a high tendency to visit health facilities after selling their harvests. Moreover, rainy and cold weather might pose difficulties for community workers to pay home visits to find presumptive TB cases. In addition, the reactivation of disease soon after an exhausting dry season harvest may explain the seasonality of TB in Ethiopia as well. That might explain why we recorded more TB cases at the end of dry season compared to the beginning of the dry season in our study, as in the study from Cameron [20].

Health care-seeking behavior may vary from season to season with domestic work such as the harvest or going to market and may extend to the number of people tested for TB [13]. Since the scale-up of the health infrastructure in Ethiopia, including the construction of new health posts and health centers and expansion of microscopy services, health care-seeking behavior and access to health services are improving. Still, the seasonal variation of patients’ visits to health facilities and then TB case notification is inevitable in Ethiopia, because 85% of the population is rural and farmers live in a climate with dry and rainy seasons [25]. Orienting health extension workers and community volunteers to the seasonality of TB could enable the detection of missed TB cases in the community, thus improving TB case fining. It is also necessary to enhance health education to heighten awareness about TB transmission during the peak quarter. Contact investigation and preventive therapy also need to be strengthened during the peak seasons.

In contrast to studies from temperate zones, the possibility that deficiency or insufficiency of vitamin D, the most widely studied reason for the seasonal variation of TB [2,4,15,16], could cause seasonal variation is less likely in our setting. However, vitamin D deficiency and lower immunity in the cooler and wet seasons cannot be ruled out [31].

We reported seasonal variation within regions. In the study regions, due to their wider geographic difference and variations in their rainy season, health care-seeking behavior might have also been different. We found 10% more variability in Amhara region compared to Oromia. The difference could be due to variations in health system functionality, socioeconomic conditions, and culture. Studies from India [17, 18] and Iran [6] indicated that seasonal variation was high in the northern part of these countries as compared to the southern due to higher differences in temperature.

There was not a significant difference in seasonal amplitude by age. Unlike our study, the research from the United States showed an increased seasonality among young children; the greatest seasonal amplitude was found among children aged less than 5 years, suggesting that TB disease is the result of recent transmission more influenced by season than by activation of latent infection [7]. In contrast, 17% seasonal amplitude among adults is greater than the 15% seasonal amplitude in under-fives in our study. This might indicate that TB disease resulting from reactivation of latent TB is influenced by season rather than by recent infection with early progression.

The forecast indicated that the trend in TB incidence will increase after a period of decrease in Oromia. This could be due to HIV [32,33], diabetes and cancers [34], and high-risk populations such as prisoners [35].

We used aggregated data and do not have information about specific sites where patients were notified or about socioeconomic condition to make detailed analyses. Still, this is one of a few studies in the tropics of sub-Saharan Africa and Ethiopia, with a large sample size that used an advanced model to describe the seasonal pattern of TB. The exponential smoothing model was applied to forecast trends, unlike the widely used ARIMA (Autocorrelation, Integration and Moving Average) model. This is because our data fitted the exponential smoothing model better than the ARIMA.

## Conclusions

TB notification varied by both regions and seasons. The peak season is from April to June and the trough, from October to December. The peak TB case notification rate corresponds to the end of the dry season, when rural residents have the resources and time to seek medical care. TB prevention and control interventions should be designed using such evidence in order to take advantage of seasonality in order to conduct high-impact interventions. Further studies are warranted to understand the effect of socioeconomic conditions, health system functions, and cultural issues that affect the seasonal patterns of TB.

## Supporting information

S1 Fig Map of the study settings, the 10 zones in Oromia and Amhara Ethiopia. **Color**: this is the map of Ethiopia, the green colored region named as”OROMIA” is one of the study regions with 5 zones. The zone are written in their respective boundaries; East Wellega, Jimma, Arsi, West and East Harerge. The other color (faded yellow) is Amhara region and indicated by “AMHARA” with its five study zones; East Gojjam, North and South Gondar, and North and South Wollo.
(PDF)

S1 Table Statistical model for the data on notified TB cases. ^a^ Best-fitting models according to stationary R-squared (larger values indicate better fit).
(PDF)

S2 Table Yearly notified TB cases based on types of TB, sex, region, and age category, 2010–2016. (PDF)
