# Supplementary figures and images for: Seasonal patterns of tuberculosis case notification in the tropics of Africa: A six-year trend analysis in Ethiopia
Source: PLoS One. 2018 Nov 26;13(11):e0207552. doi: 10.1371/journal.pone.0207552 (PMC6261032; doi:10.1371/journal.pone.0207552)

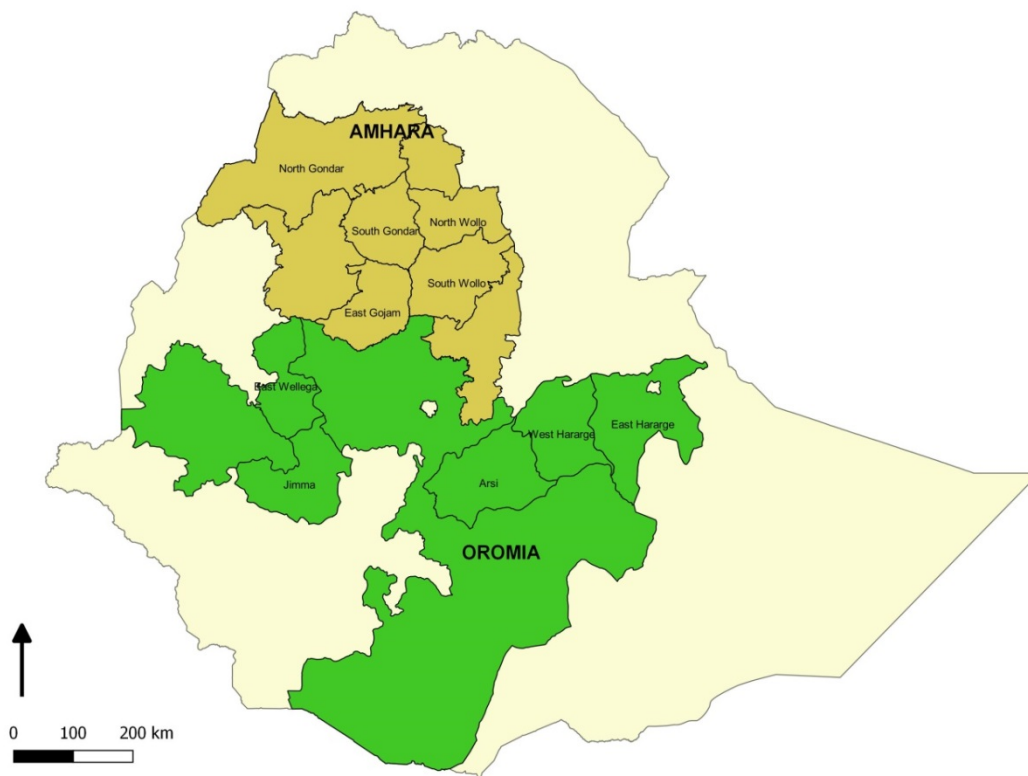

**S1 Fig. Map of the study settings, the 10 zones in Oromia and Amhara in Ethiopia**

Supplement: S1 Fig — Color: this is the map of Ethiopia, the green colored region named as”OROMIA” is one of the study regions with 5 zones. The zone are written in their respective boundaries; East Wellega, Jimma, Arsi, West and East Harerge. The other color (faded yellow) is Amhara region and indicated by “AMHARA” with its five study zones; East Gojjam, North and South Gondar, and North and South Wollo. (PDF) [file pone.0207552.s001.pdf]
