# Supplementary material for: Seasonal patterns of tuberculosis case notification in the tropics of Africa: A six-year trend analysis in Ethiopia
Source: PLoS One. 2018 Nov 26;13(11):e0207552. doi: 10.1371/journal.pone.0207552 (PMC6261032; doi:10.1371/journal.pone.0207552)
Supplement: S1 Table — a Best-fitting models according to stationary R-squared (larger values indicate better fit). (PDF) [file pone.0207552.s002.pdf]

S 1 Table. Statistical model for the data on notified TB cases

Model[a]	Number of Predictors	Model Fit Statistics	Ljung-Box Q-Test			Number of Outliers
		Stationary R-squared	Statistics	Degree of freedom (DF)	Signifiicance	
Oromia total-Model_3	0	0.665	15.769	15	0.398	0
Amhara total-Model_2	0	0.421	18.702	15	0.228	0
Total TB cases-Model_1	0	0.461	18.735	15	0.226	0
