# Supplementary material for: Seasonal patterns of tuberculosis case notification in the tropics of Africa: A six-year trend analysis in Ethiopia
Source: PLoS One. 2018 Nov 26;13(11):e0207552. doi: 10.1371/journal.pone.0207552 (PMC6261032; doi:10.1371/journal.pone.0207552)
Supplement: S2 Table — (PDF) [file pone.0207552.s003.pdf]

S2 Table. Yearly notified TB cases based on types of TB, sex, region, and age category, 2010-2016

Year	Total TB Cases	Total SS+	Total EPTB	Total SS−	Total Male	Total Female	Amhara Total	Oromia Total	Total >15 Yrs	Total <15 Yrs	Total <5 Yrs	Total 5-14 yrs
2010/11	35994	10565	13975	11454	19796	16198	16640	19354	31147	4847	642	4205
2011/12	36496	10522	14395	11580	20073	16423	18659	17837	31638	4858	636	4225
2012/13	35059	9946	13708	11404	19291	15768	17568	17491	31114	3945	884	3061
2013/14	32166	9522	12028	10616	17742	14424	14863	17303	28357	3809	798	3011
2014/15	32464	9593	12810	10060	17855	14609	15220	17244	28824	3640	998	2642
2015/16	33396	9908	13134	10355	18368	15029	15263	18133	29638	3758	1037	2722
**Grand Total**	**205575**	**60057**	**80049**	**65469**	**113126**	**92449**	**98213**	**107362**	**180715**	**24861**	**4994**	**19867**
